# Wolfram syndrome, a rare neurodegenerative disease: from pathogenesis to future treatment perspectives

**DOI:** 10.1186/s12967-019-1993-1

**Published:** 2019-07-23

**Authors:** Maria Teresa Pallotta, Giorgia Tascini, Roberta Crispoldi, Ciriana Orabona, Giada Mondanelli, Ursula Grohmann, Susanna Esposito

**Affiliations:** 1 Pharmacology Section, Department of Experimental Medicine, Università degli Studi di Perugia, Perugia, Italy; 2 Pediatric Clinic, Department of Surgical and Biomedical Sciences, Università degli Studi di Perugia, Piazza Menghini 1, 06129 Perugia, Italy

**Keywords:** Deafness, Diabetes insipidus, Optic atrophy, Type 1 diabetes, Wolfram syndrome, *WFS1*, *WFS2*

## Abstract

**Background:**

Wolfram syndrome (WS), a rare genetic disorder, is considered the best prototype of endoplasmic reticulum (ER) diseases. Classical WS features are childhood-onset diabetes mellitus, optic atrophy, deafness, diabetes insipidus, neurological signs, and other abnormalities. Two causative genes (*WFS1* and *WFS2*) have been identified. The transmission of the disease takes place in an autosomal recessive mode but autosomal dominant mutations responsible for WS-related disorders have been described. Prognosis is poor, death occurs at the median age of 39 years with a major cause represented by respiratory failure as a consequence of brain stem atrophy and neurodegeneration. The aim of this narrative review is to focus on etiology, pathogenesis and natural history of WS for an adequate patient management and for the discussion of future therapeutic interventions.

**Main body:**

WS requires a multidisciplinary approach in order to be successfully treated. A prompt diagnosis decreases morbidity and mortality through prevention and treatment of complications. Being a monogenic pathology, WS represents a perfect model to study the mechanisms of ER stress and how this condition leads to cell death, in comparison with other prevalent diseases in which multiple factors interact to produce the disease manifestations. WS is also an important disease prototype to identify drugs and molecules associated with ER homeostasis. Evidence indicates that specific metabolic diseases (type 1 and type 2 diabetes), neurodegenerative diseases, atherosclerosis, inflammatory pathologies and also cancer are closely related to ER dysfunction.

**Conclusions:**

Therapeutic strategies in WS are based on drug repurposing (i.e., investigation of approved drugs for novel therapeutic indications) with the aim to stop the progression of the disease by reducing the endoplasmic reticulum stress. An extensive understanding of WS from pathophysiology to therapy is fundamental and more studies are necessary to better manage this devastating disease and guarantee the patients a better quality of life and longer life expectancy.

## Background

Wolfram syndrome (WS) is an autosomal recessive disorder characterized by diabetes insipidus (DI), childhood-onset diabetes mellitus (DM), a gradual loss of vision caused by optic atrophy (OA), deafness (D; hence the acronym: DI DM OA D), and neurological signs. Other symptoms include bladder, bowel and temperature regulation dysfunctions, endocrinological, psychiatric, and neurological abnormalities [1]. Wolfram and Wagner first described the disease in 1938 in four of eight siblings suffering from juvenile diabetes mellitus and optic nerve atrophy [2].

Two causative genes for this genetic disorder have been identified: *Wolfram syndrome 1* (*WFS1*) and *Wolfram syndrome 2* (*WFS2*) [3, 4]. The classical form of WS is caused by autosomal recessive mutations of the *WFS1* gene, localized on human chromosome 4p, encoding a protein called wolframin. A smaller portion of patients has mutations in the *CIDS2* (CDGSH iron-sulfur domain-containing protein 2) gene, which are responsible for autosomal recessive Wolfram syndrome 2 (WS2). WS2 differs from the classical form (WS1) by the presence of bleeding, upper intestinal ulcer, defective platelet aggregation and absence of diabetes insipidus and psychiatric disorders [1, 5]. Recently, atypical forms of disease associated with one or two mutations in *WFS1*, namely dominant disease with or without DM, and recessive Wolfram like disease without DM have been reported [6–10].

Early diagnosis is mandatory in order to guarantee patients’ adequate care and follow-up based on a multidisciplinary approach, such as genetic counseling to enable families to deal with their risk and decide how to act. WS afflicts about 1 of 770,000 [11] and 1 of 500,000 in the pediatric population in the United Kingdom [12], 1 of 710,000 in the Japanese population [13], 1 of 100,000 in North America [14] and 1 of 68,000 in the Lebanese population [15]. An epidemiological study showed a prevalence of 1 in 54,478 in a district of North-Eastern Sicily. Probably, the disease prevalence is much higher in communities with high rates of consanguineous unions, as in Lebanese and Sicilian populations [16]. This syndrome is categorized as a rare specified diabetes mellitus (sub category 5A16.1, Wolfram syndrome) in the draft of International Classification of Disease (ICD-11) [6]. Due to the disease progression, with severe neurological disabilities, most affected patients die prematurely, usually from respiratory failure [1]. The typical median age of death is 39 years (with a range from 25 to 49). Careful clinical monitoring and supportive care are fundamental to relieve the debilitating symptoms [1, 17].

The aim of this narrative review is to focus on etiology, pathogenesis and natural history of WS for an adequate patient management and for the presentation of future therapeutic interventions.

## Etiology: genetic background, molecular biology and pathophysiology of the endoplasmic reticulum stress pathway

The *WFS1* gene maps to chromosome 4p16 and consists of eight exons (33.4 kb of genomic DNA). It encodes a 890 amino acid hydrophobic glycoprotein (wolframin; WFS1), composed of nine transmembrane segments and localized primarily in the membrane of endoplasmic reticulum (ER) [3]. Secondary structure predictions identify three structural domains, namely, a hydrophobic central domain comprising 9–10 membrane-spanning segments flanked by two hydrophilic domains at the N- and C-terminus. WFS1 is highly expressed in the brain tissue, pancreatic β-cells, heart, lung, and placenta [18].

Currently, over 200 distinct mutations have been identified in WS patients, most of which are located in exon 8 [19], particularly, in the region that encodes for the transmembrane and C-terminal domain of the protein [3, 20, 21]. It has been established that WFS1 is important for maintaining ER homeostasis, but the mechanisms are still unclear [22].

Because of the large number of variants of the *WFS1* gene identified so far and the small size of patient cohorts, it is still difficult to correlate WS clinical features with the mutations found in this gene. An important effort to establish a reliable genotype–phenotype correlation was performed by de Heredia et al. [23], which analyzed both genetic and clinical data of 412 patients with WS published since 1998. According to this analysis and the recent review from Rigoli et al. [5], it was possible to identify a correlation between the *WFS1* gene mutation and the effect on the protein. They also evaluated the disease progression rate and the main clinical manifestations in WS (Table 1). However, atypical forms of disease associated with one or two mutations in *WFS1* appear intriguing since classical inactivating mutations cannot be the mechanisms.

**Table 1:** Clinical manifestations of WS

Major clinical signs	Other common clinical signs
Diabetes mellitus^a^ [40] Average age of diagnosis 6 years	Urinary tract problems and renal dysfunction [13, 38] Neurogenic bladder Bladder incontinence Urinary tract infection Average age of diagnosis 12–20 years
Optic atrophy^a^ [40] Average age of diagnosis 10–11 years	Psychiatric symptoms [52] Depression Psychosis Panic attacks Sleep abnormalities Mood swings
Diabetes insipidus [13, 38] Average age of diagnosis 14–15 years	Neurological manifestation/autonomic dysfunction [12, 38] Central apnea Dysphagia Areflexia Epilepsy Decreased ability to taste and detect odors Headache Orthostatic hypotension Hypothermia, hyperpyrexia Gastroparesis, constipation
Sensorineural hearing loss [13, 38] Average age of diagnosis 16 years	Endocrine disorders [12, 17] Hypogonadism Deficient growth hormone secretion Deficient corticotropin secretion Delayed menarche in female
Neurological manifestation [38]: ataxia Average age of diagnosis 15 years	
Atypical forms [6–8]	Dominant disease with or without diabetes mellitus and recessive Wolfram like disease without diabetes mellitus
Severe gastrointestinal ulcers, bleeding and defective platelet aggregation^b^ [12]	

^a^Required for diagnosis

^b^Associated with WFS 2

The majority of mutations has an autosomal recessive mode of transmission. Moreover, in the literature, autosomal dominant mutations have been described as responsible for WS-like disease [9, 10]. Therefore, the WFS1-related non-syndromic low frequency sensorineural hearing loss (LFSNHL), also known as DFNA6/14/38 LFSNHL, is characterized by autosomal dominant transmission, with missense mutations in exon 8. Clinically, it is defined by LSFNHL, psychiatric illness and DM [24–26].

A second and rarer form of WS is WS2, caused by a homozygous mutation in the zinc-finger protein named as endoplasmatic reticulum intermembrane small protein (ERIS) encoded by the *WFS2* (*CISD2*) gene, mapping to 4q22-q24 [4]. ERIS plays an important role in ER, mitochondria membrane integrity, and in the functional cross-talk between these two cellular compartments [27].

**Fig. 1:**
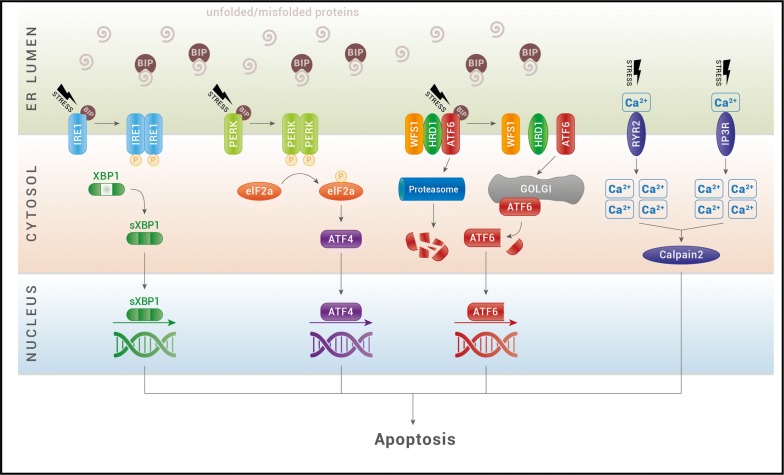
The ER stress pathway. Under situations of stress, unfolded and misfolded proteins accumulate and recruits BIP to the ER lumen. BIP dissociates from the ER stress sensors IRE1α (inositol-requiring protein 1), ATF6 (activating transcription factor 6) and PERK [protein kinase RNA (PKR)-like ER kinase] and leads to their activation. Upon dimerization and autophosphorylation, IRE1 induces the splicing of XBP1 mRNA for translation of the transcription factor spliced XBP1 protein (sXBP1). XBP1s translocates to the nucleus and controls the transcription of ER-resident chaperones, components of the ERAD machinery and genes involved in lipogenesis. Activated PERK causes the phosphorylation of eukaryotic initiation translation factor 2α (eIF2*α*), which increases production of activating transcription factor 4 (ATF4). ATF4 then translocates to the nucleus and induces the transcription of many genes required for ER quality control. Activated ATF6 translocates to the Golgi, where it is processed by S1P and S2P proteases. The cleaved-off cytoplasmic domain functions as a transcription factor and induces the expression of ER chaperones and XBP1. ATF6 activity is inhibited by the WFS1 protein, that through the E3 ubiquitin ligase HRD1, is responsible of ATF6 ubiquitin-mediated proteasomal degradation. ER calcium channels, ryanodine receptor (RyR) and inositol triphosphate receptor (IP3R), control efflux of calcium (Ca^2+^) from the ER to the cytosol. Under ER stress activation, these receptors increase the levels of cytosolic calcium and activate the calcium-dependent protease, calpain-2, which promotes cellular apoptosis

ER is the cellular organelle with an essential role for cell survival. It is the most important storage for Ca^2+^ ions and is responsible for the correct folding and posttranslational modification of secretory proteins, cell surface receptors, and integral membrane proteins [22]. The sensitive folding environment of the ER can be perturbed by physiological processes, such as post-prandial insulin biosynthesis that requires a great biosynthetic activity from the ER in response to food uptake, or pathological processes like viral infection, toxins, cytokines, and mutant protein expression. In these situations, if ER exceeds its folding capacity, the balance of this delicate system can be disrupted with an accumulation of unfolded/misfolded protein inside the ER lumen that cannot be processed through the secretory pathway. As a consequence, cells undergo a condition defined as ER stress, which activates a network of signaling pathways called “unfolded protein response” (UPR). UPR’s primary function is to mitigate ER stress, and generate proteins for survival. Under these conditions, ER stress is beneficial. However, under pathological conditions with chronic and high ER stress, UPR is unable to reduce stress and retrieve homeostasis. Therefore, the cell undergoes irreversible damage which leads to apoptosis [27–29] (Fig. 1).

UPR performs its role by activating three signaling proteins: inositol-requiring protein 1 (IRE1), protein kinase RNA (PKR)-like ER kinase (PERK) and activating transcription factor 6 (ATF6). Activation of these transducers can culminate in both survival-adaptive and death responses. Under physiological conditions, the ER chaperones BIP (binding immunoglobulin protein) binds their luminal domains maintaining them in an inactive state. When unfolded/misfolded proteins accumulate in the ER lumen, BIP is released from these complexes to assist with the folding of accumulated proteins [30, 31].

During physiological ER stress, the transmembrane IRE1 protein oligomerizes and autophosphorylates its cytosolic domain [32]. As a consequence, the IRE1 RNase domain promotes an atypical splicing of X-box—binding protein 1 (XBP-1) mRNA to form a transcriptionally active mRNA, named sXBP-1. This is converted into active XBP-1, a transcription factor that traslocates to the nucleus and upregulates a variety of UPR target genes, including ERAD (ER-assisted degradation) components as well as foldases such as protein disulfide isomerase (PDI) and BIP, in order to restore protein homeostasis and promote cytoprotection. Under pathological conditions, IRE1 is hyperactivated and activates apoptotic pathways by recruiting TRAF2 (TNF receptors-associated factor 2) and phosphorylating the apoptosis signal-regulating kinase (ASK1). ASK1, in turn, phosphorylates the c-Jun N-terminal kinase (JNK), which regulates protein member of the B-cell lymphoma (BCL) family to induce apoptosis [30, 33]. IRE1 has also a role in insulin biosynthesis: under high glucose conditions, it enhances pro-insuline biosynthesis promoting homeostasis in β-cells [33, 34].

In case of ER stress, the transmembrane PERK protein is released from BIP, dimerizes, undergoes trans-autophosphorylation and directly phosphorylates the eukaryotic initiation translation factor 2α (eIF2α). eIF2α reduces ER biosynthetic activity by promoting and encouraging this way of adaptation, but it also increases the translation of certain mRNAs, such as those encoding for ATF4 and the apoptosis antagonizing transcription factor (AATF). ATF4 transcriptionally induces genes involved in amino acid transport and metabolism, glutathione biosynthesis, and antioxidant responses, but also induces ATF3 and CHOP [35]. AATF is a recently discovered anti-apoptotic mediator that promotes cell survival. Under unresolvable ER stress conditions, a continuous stimulation of these factors induces cell death by the expression of the CHOP factor CHOP (CCAAT/enhancer-binding protein homologous protein), which regulates the expression of BCL2 family members.

ATF6 is the third master regulator of the UPR. Under ER stress conditions, BIP dissociation drives ATF6 translocation to the Golgi apparatus, where it is cleaved by proteases into a cytosolic active transcription factor. The processed form of ATF6 translocates to the nucleus where it upregulates transcriptionally ER homeostatic effectors to enhance protein folding, processing, and degradation capacity. However, its hyperactivation leads to cells dysfunction and death. ATF6 regulates also genes involved in lipid biosynthesis, ERAD and insulin gene expression, and its hyperactivation seems to suppress the transduction of these genes [36, 37].

WFS1 seems to be a negative regulator of the UPR pathway. In healthy cells, under physiological stress, it prevents ATF6 activation by recruiting ATF6 to HRD1 (HMG-CoA reductase degradation protein 1), an E3 ligase, for ATF6 ubiquitination and proteasomal degradation (Fig. 1). In WS patients, ATF6 is hyperactivated and, in turn, constantly activates genes that promote cellular apoptosis (CHOP, ATF4, BIP, and sXBP1) and decrease insulin gene expression. WFS1 expression has been shown to be induced during insulin secretion, suggesting that WFS1 is an important component of proinsulin folding and processing in the ER of the β-cell [38–40]. Other studies suggested that WFS1 can also regulate Ca^2+^ signal transduction processes, thus influencing the storage of cellular ER calcium levels and, consequently, cells apoptosis [28, 41]. WFS1-deficient β-cells and neurons have reduced Ca^2+^ in ER and increased cytosolic Ca^2+^ levels, a condition that leads to activation of the Ca^2+^-dependent cysteine protease calpain and cell death [42, 43]. It has been also reported that WFS1 binds to and modulates the function of sarco/endoplasmic reticulum Ca^2+^-ATPase (SERCA), which is one of the major proteins required for ER calcium homeostasis in β cells [44, 45]. Moreover, WFS1 probably plays an important role during embryogenesis [46].

## Natural history, clinical manifestations, and clinical management

Although atypical dominant and recessive cases have been described [6–8], the common features of WS are DM, OA, central DI, sensorineural D, urinary tract problems, and progressive neurologic difficulties [1]. The order of onset of clinical symptoms and the natural history of WS have been the objective of many studies but, due to the molecular complexity of the syndrome and the small proportion of patients, a clear genotype–phenotype correlation is still difficult to establish [26, 46–48]. Table 1 summarizes the main clinical manifestations in WS, including those reported in atypical cases. A study of 412 patients with WS showed that 98.21% had DM, 82.14% had OA, 48.21% had D, 37.76% had DI, whereas urological manifestations and neurological symptoms were present in 19.39% and 17.09%, respectively. This syndrome is characterized by high mortality and morbidity and the main cause of death in affected patients is represented by respiratory failure. Moreover, two peaks of higher frequency of death—one at 24 and one at 45 years of age—can be observed, with a median age around 30 years [23].

Non-autoimmune and non-HLA-linked insulin-dependent diabetes is generally the first symptom diagnosed around age 6 (range 3 weeks to 16 years old). Compared to type 1 diabetes (T1D), ketoacidosis is rare, patients have a longer duration of the remission period, lower insulin requirements and HbA1c levels. However, due to neurologic dysfunctions caused by a perturbation of ER function, episodes of severe hypoglycemia are more frequent. A smaller frequency of microvascular complications and the slower progression of the same could be related to a persistence of residual insulin secretion and total insulin deficiency, which is not as quick as in T1D. Degeneration of pancreatic β-cells, where WFS1 is highly expressed, determines insulinopenia. However, in literature, cases of co-occurrence of WS and T1D have been reported [5, 49, 50].

OA appears at an average age of 11 years (6 weeks to 19 years old) [8]. OA is a criterion required for the diagnosis of WS and is characterized by a progressive decrease of visual acuity and color vision defect. Ophthalmologic manifestations include cataracts, abnormal papillary light reflexes, nystagmus, glaucoma, and pigmentary maculopathy [5, 8, 51]. Experimental studies in monkey and mouse retinas demonstrated that WFS1 is expressed in retinal ganglion cells, cells of the inner nuclear layer, photoreceptors, and in glial cells of the proximal portion of the optic nerve. Mitochondrial alterations, recently demonstrated in WS, would justify the appearance of pigmentary maculopathy, typical of the mitochondrial disorders themselves, including the Kearns–Sayre syndrome and neuropathy, ataxia, retinitis pigmentosa (NARPS) [51]. In literature, few cases of WS associated with any type of retinal pigmentary changes have been described. Nine out of 91 patients studied by Cremers et al. had retinal pigment dystrophy [52, 53]. Gunn et al. found a patient with “perimacular granularity”, whereas, of the 45 cases analyzed by Barret et al. none had pigmentary retinal alterations [54, 55]. A new ocular anomaly, i.e., microspherophakia, was observed in two sisters and associated with a novel WFS1 in-frame delection (c.1525_1539 homozygous deletion in exon 8). Both siblings also presented cataracts, glaucoma, and OA [55]. Zmyslowska et al. [56] by using high-definition OCT and MRI parameters, showed that retinal thickness is lower on average in subjects with WS than patients with T1D or healthy subjects. Furthermore, authors in this study underlined the importance of high-definition OCT in combination with MRI as tools for an adequate monitoring over time of the disease as well as for the evaluation of therapeutic trials in patients with WS.

DI appears at an average age of 14 years (3 months to 40 years old). Approximately 73–75% of patients present partial cranial DI, whose diagnosis is often delayed. In all subjects with T1D, tests for deafness, neurological defects, ocular anomalies, and concentration ability of urine must be performed. First line treatment is intranasal or oral administration of desmopressin and, generally, most patients with WS respond well to therapy [5, 18].

Over time, the focus on variability of the beginning and progression of symptomatology in WS has been growing. Many studies suggest hearing impairment being usually diagnosed in the second or third decade of age [11]. In particular, hearing impairment develops at an average age of 16 years (range 5 to 39 years old) and manifests in approximately 62% of patients [12]. By increasing age, hearing impairment is more pronounced than in other patients with hypoacusia, probably as a consequence of progressive central nervous system degradation [57]. Compared to the gender, only some studies have shown a greater alteration in the female sex [11, 53, 57]. Audiometry tests every year or two and also an auditory brainstem response (ABR) is used for evaluating the natural course of WS and efficacy of treatment. An adequate clinical monitoring is mandatory for appropriate intervention, also considering the numerous described cases of patients with early onset hearing impairment from birth to 3 years of age 38. Hearing aids and cochlear implants can be used as therapeutic tool [58].

Some studies agree on the frequency of neurological signs, which are ranked third after DM and OA. Neurological complications involve 62% of WS patients, appearing at an average age of 16 years (mean age 30 years old, range 5–44 years old) [11]. Nevertheless, Chaussenot et al. [59] found that the onset of neurological symptoms is often much earlier than previously reported. Moreover, Heredia et al. showed that neurological abnormalities develop at a mean age of 23 with two peaks, at 13 and 30 years of age [23, 26].

Cerebellar ataxia is the most common neurologic complication [11]. Other signs are dysarthria, dysphagia, areflexia, epilepsy (absence, myoclonic epilepsy or tonic–clonic seizures), nystagmus, and decreased ability to taste and detect odors. Moreover, patients may present orthostatic hypotension, anhidrosis, hypohidrosis or hyperhidrosis, gastroparesis, hypothermia or hyperpyrexia, constipation (typical of autonomic neuropathy). Headaches have also been described [23, 26].

Common causes of mortality are respiratory failure or dysphagia, as consequence of brain stem atrophy [11, 12]. Dysphagia can be responsible for aspiration pneumonia. For diagnosis of central apneas due to brain stem involvement, polysomnography and overnight oximetry tests are important, also because in some cases tracheostomy is necessary [11, 12].

Other features are severe depression, psychosis, sleep abnormalities, impulsive verbal, and physical aggression. In general, psychiatric illness, characterized by anxiety, panic attacks, and mood swings, appear belatedly and specialist support is required [60]. Studies reported high expression of WFS1 in the limbic system including amygdaloid areas, hippocampal region, olfactory tubercles, and superficial layer of piriform allocortex [52]. Lodha et al. [61] described a case of a 16-year-old young girl with WS affected by a depressive disorder who presented suicide attempt. Thus, a multidisciplinary approach, including specific psychotherapy, is necessary to avoid disastrous consequences. WS patients generally maintain a normal cognitive performance. Nevertheless, a study conducted with a group of 59 subjects showed that cognitive disability involves 32% of patients themselves, after cerebellar ataxia and peripheral neuropathy [48].

Urinary tract problems are commonly reported in WS. Upper tract dilatation, urinary incontinence, and recurrent infections are related to neurogenic bladder. Up to 90% of patients can be involved, with a median age of onset at 20 years of age and three peaks, respectively at 13, 21 and 33 years of age [62]. Clinical, instrumental and laboratory controls of renal function, with the measurement of post-void residual urine volume by ultrasound and urodynamic testing, are recommended. Therapy for bladder dysfunction is composed by anticholinergic and clean intermittent catheterization [48]. In literature, cases with chronic renal failure have been reported. Some members affected by the syndrome of a Turkish family were found to suffer renal and retinal complications with rapid progression [62]. Another case described a 14-year-old adolescent patient, developing end-stage renal failure requiring hemodialysis [63–65].

Additional abnormalities in WS include hypogonadism (primary and secondary), frequently in male patients, and delayed menarche in females with normal ovarian activities [11]. Short stature and deficient growth hormone (GH) secretion, or even deficient corticotropin secretion, have been described. Growth must be monitored so that GH can be used as marker of response to treatments in case of severe growth retardation. In fact, steroid supplementation may be necessary for some patients during a severe infection [5, 15].

Gastrointestinal disorders as bowel dysmotility, gastroparesis, and bowel incontinence have been reported [59]. Albeit rarely, cases of valvular heart disease or cardiac anomalies have also been found, such as Tetralogy of Fallot and pulmonary valve stenosis [24, 66, 67].

## Diagnosis

Usually, history and clinical manifestations, based on the presence of optic nerve atrophy after diagnosis of diabetes mellitus under the age of 16, generate suspicion in the clinician [55]. However, manifestation of visual abnormalities accompanying DM in patients with WS may lead to a misdiagnosis of T1D with diabetic retinopathy in some children and adolescents, which may result in delayed recognition and underestimation of the prevalence of WS in the childhood population. Zmylowska et al. in a recent study, observed the delay between diagnosis of OA and referral genetic analysis to be 7 years on average [1, 68].

Genetic tests are necessary to confirm the diagnosis and, in relation to the molecular complexity of the pathology, the interpretation of results requires specialized knowledge. In addition, exome sequencing and genome sequencing-based diagnostic methods for this entity and for Wolfram-related disorders have been created. Early diagnosis is imperative to enable proper prognostication, prevent complication, and start available treatments. An efficacious follow-up includes regular neurological, ophthalmologic and psychiatric consultations, audiometry as well as endocrine check-ups. An early diagnosis is also important from the perspective of prevention. A careful assessment of patient’s siblings is recommended even if they are asymptomatic. A thorough genetic counselling and possibly prenatal diagnosis can in fact enable the affected families to cope with the risk of mitochondrial disorders, Friereich ataxia, and Bardet–Bield disease.

## Treatment and new perspectives

The main goals for WS treatment are representing by the stop of progression of the disease and replacement of damaged tissues, such as those including pancreatic β-cells and retinal cells [1]. In order to achieve the first objective, medical drugs should be directed to the maintenance of ER homeostasis and thus of calcium homeostasis, redox regulation, and protein folding [1, 69, 70]. In fact, WS is considered an important prototype of human ER disease and, in the last few years, clinical and genetic studies have in fact elucidated the consequences of ER stress. Currently, specific and effective therapy is not available yet and drug repurposing represents the best therapeutic option (Table 2). This means that drugs approved for other diseases by regulatory agencies, such as Food and Drug Adminstration (FDA; United States) or European Medicine Agency (EMA), could be used in WS patients [1, 5].

**Table 2:** Drug proposed to treat WS

Compound	Target/mechanism of action	Clinical trial status in WS	References
4-Phenylbutyric acid (PBA) and tauroursodeoxycholic acid (TUDCA)	Chemical chaperones: stabilize protein conformation during folding, ameliorate trafficking of mutant proteins, suppress unfolded protein aggregation		[71]
Dantrolene	Blocks ryanodine receptor in the ER membrane: stabilize ER calcium level by suppressing the efflux of calcium from ER to cytosol	Clinical trial of dantrolene sodium in pediatric and adult patients with WS, ClinicalTrials.gov: NCT02829268	[28, 72, 74]
Pioglitazone	Inhibits inositol triphosphate (IP3R)-mediated release of calcium from the ER		[45, 75–78]
Rapamycin	Reduces cytoplasmic calcium by a mechanism similar to pioglitazone		[76]
Carbachol	Muscarinic receptor 3 (M3) agonist: mobilizes intracellular calcium stores and potentiates glucose-stimulated insulin secretion		[79, 80]
Liraglutide, exenatide, semaglutide	Glucagon-like peptide-1 receptor (GLP-1R) agonists. They activate PERK-ATF4 pathway and interfere with the ER unfolded protein response		[28, 81–86]
Sitagliptin, vildagliptin, gemigliptin	Inhibitors of dipeptidyl peptidase-4 (DPP-4), an enzyme that deactivates GLP-1, thus also increasing GLP-1 levels		[71]
Salubrinal	Selective inhibitor of the eIF2α phosphatase		[87, 88]
Valproate acid (VPA)	Promotes the expression of both WFS1 and ER chaperones and attenuates ER-induced apoptosis	Efficacy and safety trial of sodium valproate in pediatric and adult patients with WS, ClinicalTrials.gov: NCT03717909	[89–94]

A therapeutic strategy is based on the use of chemical chaperones, a class of molecules which assist protein folding in the ER. Currently, two chemical chaperones have been approved by FDA, namely, 4-phenylbutyric acid (PBA) and tauroursodeoxycholic acid (TUDCA). These drugs seem to preserve β-cells functions by reducing stress in the ER compartment and cell death and they also decelerate neurodegeneration in WS patients, probably by acting via the same mechanism in neuronal cells. PBA has been shown to stabilize protein conformation during folding, ameliorate trafficking of mutant proteins, and to restore the normal pattern of insulin secretion [71].

In WS, the depletion of ER calcium and subsequent activation of calpain may play a role in β-cell death and neurodegeneration [42]. Thus, because modulation of cellular calcium levels may prevent calcium-mediated ER stress and cell death, another target therapy for WS could be represented by FDA-approved compounds that may stabilize ER calcium level. In particular, it has been demonstrated that dantrolene, by blocking the ryanodine receptor (RyR) localized to the ER membrane, suppresses efflux of calcium from ER to cytosol and leads to the integrity preservation of β- and neural cells. Dantrolene is a muscle relaxant drug prescribed for multiple sclerosis, cerebral palsy, and malignant hyperthermia [72]. Its main side effect is hepatotoxicity, which may be do to either asymptomatic elevation of liver enzymes and/or bilirubin or, more severe, fatal and nonfatal liver inflammation, even with sporadic short-term use [73]. The risk of liver inflammation is associated with duration of treatment and daily dose. Recently, it has been suggested that doses < 200 mg/day may be safely used in patients without co-existing liver dysfunction or co-ingestion of hepatotoxic medications. There is also a phase 1 clinical trial currently investigating the safety of dantrolene long-term use in WS patients (A Clinical Trial of Dantrolene Sodium in Pediatric and Adult Patients with Wolfram Syndrome, ClinicalTrials.gov, NCT02829268) [28, 74]. Given that WFS1 can bind SERCA, a protein that regulates calcium homeostasis, a therapeutic strategy could be the development of molecules targeting SERCA in order to increase and maintain high calcium levels in ER.

Finally, another possible target to control the release of calcium from the ER to the cytoplasm could be the calcium channel receptor activated by inositol triphosphate (IP3R) [45]. In 2009, pioglitazone, a drug already used in the treatment of T2D, was tested in WFS1 knockout mice, and results showed that mice were protected from pancreatic β-cell death, thus resulting to be more resistant to the development of diabetes [75]. Pioglitazone belongs to the category of thiazolidinediones that they may also act by inhibiting IP3R-mediated release of calcium from the ER [76]. However, the well-known adverse effects (heart failure, osteoporosis, and bladder cancer) make them not attractive drugs [77, 78]. Rapamycin, an immunosuppressive drug, is thought to reduce cytoplasmic calcium by a mechanism similar to pioglitazone but, similarly, has side effects and is expensive, features that make it a less promising therapeutic option to investigate. Furthermore, a strategy to reduce cytosolic calcium, because of negative effects on mitochondrial dynamics, is still controversial [46].

Activation of the muscarinic pathway has been recently demonstrated to be a promising target to manage diabetes in WS patients. Indeed, a study conducted in WFS1-deficient mice showed that carbachol, a muscarinic receptor 3 (M3) agonist, potentiates glucose-stimulated insulin secretion [79]. The rationale of this study was to mimic the effect of acetylcholine, the neurotransmitter released from parasympathetic nerve endings or pancreatic α-cells. Acetylcholine binding to M3 muscarinic receptors leads to the generation of IP3 and diacylglycerol. IP3 binds to IP3Rs on the ER surface, mobilizes intracellular calcium stores, and increases cytoplasmatic Ca^2+^ levels, thus potentiating glucose-stimulated insulin secretion [80].

Glucagon-like peptide-1 receptor (GLP-1R) agonists have a potential role as therapeutic agents in patients with WS [28, 81, 82]. In 2006, Yusta et al. demonstrated that treatment with GLP-1R agonists such as exenatide, (an incretin mimetic drug already used in the treatment of T2D) was associated with a 70% reduction in daily insulin dose and better glycemic control in patients with type 2 WS [28, 82]. In the current year, Kondo et al. [81] have shown that liraglutide (a long acting agonist of GLP-1R) improves patient’s glycemic control and reduces the daily insulin dose by 20%. Glucagon-like peptide (GLP) is a peptide secreted from intestinal L cells after meals and is endowed with numerous physiological actions, including decreasing pancreatic β-cell apoptosis mediated by ER stress and enhancement of β-cell growth and survival through the activation of the PERK-ATF4 pathway. Moreover, GLP-1 exerts neuroprotective effects in both central and peripheral nervous system [83]. However, the use of GLP-1R agonists is still controversial, since two clinical trials for T2D reported that progression of diabetic retinopathy is neutral with liraglutide or worsens when compared with placebo in the case of semaglutide [84–86]. Another strategy would be the use of inhibitors of dipeptidyl peptidase-4 (DPP-4) (i.e., sitagliptin, vildagliptin, gemigliptin), an enzyme that deactivates GLP-1, thus also increasing GLP-1 levels65. Because one of the mechanisms by which ER stress will promote apoptosis is the phosphorylation of eIFα, salubrinal, a selective inhibitor of the dephosphorylation of eIF2, has been tested. However, discrepancies concerning the pharmacological actions of salubrinal have been reported. In fact, whereas on one hand the drug protects PC12 neuronal cells from ER stress [87], on the other, it potentiates fatty acid-induced ER stress and apoptosis in *β*-cells [88].

A research project, currently in progress at Birmingham University, has the aim of studying valproate acid (VPA) as a novel, repurposed drug treatment for neurodegeneration and diabetes in WS. Several studies reported that VPA is indeed neuroprotective, by exerting neurotrophic effects and promoting neurite outgrowth via the inhibition of ER stress-induced apoptosis [89, 90]. VPA promotes the expression of both WFS1 and ER chaperones and attenuates ER-induced apoptosis after both ischemia/reperfusion (I/R) injury in retina and in a model of diabetic nephropathy [91–93]. This anti-epileptic drug has been designated as an orphan drug for the treatment of WS and recently it started to be studied in a phase 2 clinical trial in adult and pediatric patients with WS (ClinicalTrials.gov: NCT03717909) [94].

A great challenge in WS treatment is regenerative and gene therapy that may lead to replacement of damaged tissues, such as those including pancreatic β-cells and retinal cells. To this purpose, several studies are ongoing in Urano’s laboratory, that is one of the most important worldwide center on WS [1]. In particular, the main aim is to obtain induced pluripotent stem cells (iPS) from skin cells of patients with WS, to be differentiated into neurons, retinal cells and β-cells and thus to be used for transplantation [71]. Gene therapies based on adeno-associated virus and Clustered Regularly Interspaced Short Palindromic Repeats (CRISPR) technology are intensively being studied to correct *WFS1* mutations [28, 95]. Gene therapy is also investigated to induce production of mesencephalic astrocyte-derived neurotrophic factor (MANF) in WS patients. In fact, MANF is a regeneration factor produced by astrocytes that can prevent cell death and activate the proliferation of remaining β-cells, neurons, and retinal ganglion cells by leveraging the natural ability of the human body to regenerate damaged tissues [96, 97].

All these findings demonstrate the possibility of a pre-clinical approach to treat WS by both drug repurposing and gene therapy, and encourage further studies for a specific treatment of WS patients, i.e., by mainly targeting and solving the ER stress.

## Conclusion

WS, a rare devastating disease that affects different organs and systems, requires a multidisciplinary approach in order to be successfully treated. A prompt diagnosis decreases morbidity and mortality through prevention and treatment of complications. Being a monogenic pathology, WS may represent a perfect model to study the mechanisms of ER stress and how this condition leads to cell death, in comparison with other prevalent diseases in which multiple factors interact to produce the disease manifestations. However, the fact that we do not know the mechanisms behind the spectrum of mono- and biallelic organ manifestations and that mitochondrial dynamics abnormalities have been shown in WS may indicate the existence of a higher level of complexity than a monogenic disease. Moreover, similarly to many mitochondrial diseases, the complexity could also reside in the occurrence of a cascade of events, whose outcome could be unpredictable. WS is also an important disease prototype to identify drugs and molecules associated with ER homeostasis. Evidence indicates that specific metabolic diseases (type 1 and type 2 diabetes), neurodegenerative diseases, atherosclerosis, inflammatory pathologies and also cancer are closely related to ER dysfunction. An extensive understanding of the molecular biology and pathophysiology of WS may be thus fundamental for an adequate patient management and comprehensive genetic counseling to achieve future effective therapy.

## Data Availability

The data and materials used are included in the review.

## Abbreviations

AATF: apoptosis antagonizing transcription factor
ABR: auditory brainstem response
ASK1: apoptosis signal-regulating kinase
ATF6: activating transcription factor 6
BCL: B-cell lymphoma
BIP: binding immunoglobulin protein
CIDS2: CDGSH iron-sulfur domain-containing protein 2
CRISPR: Clustered Regularly Interspaced Short Palindromic Repeats
D: deafness
DI: diabetes insipidus
DM: diabetes mellitus
DPP-4: dipeptidyl peptidase-4
EMA: European Medicine Agency
ER: endoplasmic reticulum
ERAD: ER-assisted degradation
eIF2a: eukaryotic initiation translation factor 2α
FDA: Food and Drug Adminstration
GLP-1R: glucagon-like peptide-1 receptor
I/R: ischemia/reperfusion
IP3R: inositol triphosphate
iPS: induced pluripotent stem cells
IRE1: inositol-requiring protein 1
JNK: Jun N-terminal kinase
M3: muscarinic receptor 3
MANF: mesencephalic astrocyte-derived neurotrophic factor
NARPS: neuropathy, ataxia, retinitis pigmentosa
OA: optic atrophy
PBA: 4-phenylbutyric acid
PDI: protein disulfide isomerase
PERK: protein kinase RNA-like ER kinase
RyR: ryanodine receptor
T1D: type 1 diabetes
TRAF2: TNF receptors-associated factor 2
TUDCA: tauroursodeoxycholic acid
UPR: unfolded protein response
VPA: valproate acid
XBP-1: X-box—binding protein 1
WS: Wolfram syndrome
WFS1: Wolfram syndrome 1
WFS2: Wolfram syndrome 2
